# Richness in Functional Connectivity Depends on the Neuronal Integrity within the Posterior Cingulate Cortex

**DOI:** 10.3389/fnins.2017.00184

**Published:** 2017-04-07

**Authors:** Anton R. Lord, Meng Li, Liliana R. Demenescu, Johan van den Meer, Viola Borchardt, Anna Linda Krause, Hans-Jochen Heinze, Michael Breakspear, Martin Walter

**Affiliations:** ^1^Department of Behavioral Neurology, Leibniz Institute for NeurobiologyMagdeburg, Germany; ^2^Clinical Affective Neuroimaging Laboratory, Otto-von-Guericke UniversityMagdeburg, Germany; ^3^QIMR Berghofer Medical Research InstituteBrisbane, QLD, Australia; ^4^Department of Neurology, Otto-von-Guericke UniversityMagdeburg, Germany; ^5^Department of Cognition and Emotion, Netherlands Institute for Neuroscience, An Institute of the Royal Academy of Arts and SciencesAmsterdam, Netherlands; ^6^Department of Psychiatry and Psychotherapy, Otto-von-Guericke UniversityMagdeburg, Germany; ^7^Center of Behavioral Brain Sciences, Otto-von-Guericke UniversityMagdeburg, Germany; ^8^Metro North Mental Health Service, Royal Brisbane and Women's HospitalBrisbane, QLD, Australia; ^9^Department of Psychiatry, Eberhad Karls University TuebingenTuebingen, Germany

**Keywords:** fMRI, MRS, graph metric, aged, cortical thickness

## Abstract

The brain's connectivity skeleton—a rich club of strongly interconnected members—was initially shown to exist in human structural networks, but recent evidence suggests a functional counterpart. This rich club typically includes key regions (or hubs) from multiple canonical networks, reducing the cost of inter-network communication. The posterior cingulate cortex (PCC), a hub node embedded within the default mode network, is known to facilitate communication between brain networks and is a key member of the “rich club.” Here, we assessed how metabolic signatures of neuronal integrity and cortical thickness influence the global extent of a functional rich club as measured using the functional rich club coefficient (fRCC). Rich club estimation was performed on functional connectivity of resting state brain signals acquired at 3T in 48 healthy adult subjects. Magnetic resonance spectroscopy was measured in the same session using a point resolved spectroscopy sequence. We confirmed convergence of functional rich club with a previously established structural rich club. N-acetyl aspartate (NAA) in the PCC is significantly correlated with age (*p* = 0.001), while the rich club coefficient showed no effect of age *(p* = 0.106). In addition, we found a significant quadratic relationship between fRCC and NAA concentration in PCC (*p* = 0.009). Furthermore, cortical thinning in the PCC was correlated with a reduced rich club coefficient after accounting for age and NAA. In conclusion, we found that the fRCC is related to a marker of neuronal integrity in a key region of the cingulate cortex. Furthermore, cortical thinning in the same area was observed, suggesting that both cortical thinning and neuronal integrity in the hub regions influence functional integration of at a whole brain level.

## Introduction

Functionally, human brains are known to exhibit a highly organized structure (Meunier et al., 2010). Sub-networks within the brain have been identified which activate while performing various tasks related to visual stimuli (Calhoun et al., 2001), action (Karni et al., 1995), cognitive tasks (Buckner et al., 1996), or at rest (Damoiseaux et al., 2006). Brain regions involved in each of these tasks organize into distinct communities (Meunier et al., 2009). Underlying these segregated communities is a set of highly inter-connected regions facilitating functional connectivity between communities (Sporns et al., 2007). Typically highly influential regions are both structurally (van den Heuvel and Sporns, 2011) and functionally (van den Heuvel et al., 2009) interconnected, forming the so called rich club (Colizza et al., 2006). Despite advancements in imaging analysis techniques regarding graph theoretical analysis for functional MRI, there is currently little knowledge of the biochemical and biological neurotransmitter underpinnings of graph outcome metrics.

The rich club in human brains was first characterized structurally using diffusion tensor imaging (DTI) (van den Heuvel et al., 2013; Collin et al., 2014a), highlighting a consistent set of key regions. High resolution DTI studies have identified a myriad of rich club regions including frontal-temporal, anterior and posterior midline regions, and posterior cingulate cortex (van den Heuvel and Sporns, 2011). Functionally, there are few studies exploring the rich club in human brains at rest. One such study that found a significant enrichment of the fRCC in healthy adults when compared to adolescents (Grayson et al., 2014). While rich club like properties have been observed in both structural and functional modalities of brain imaging, fundamental underlying differences in imaging techniques and their subsequent analysis preclude meaningful direct comparison of the two. Most importantly, resting state functional connectivity is susceptible to the transitive property, particularly between highly correlated regions (Langford et al., 2001), an effect that structural imaging does not suffer from.

Recent studies have identified a number of regions which typically enter their active state during periods of rest with eyes closed (Zhang and Raichle, 2010), commonly known as the default mode network (DMN). Within the DMN the posterior cingulate cortex (PCC) plays an important role, being directly structurally connected to both the medial temporal lobe (MTL) and the medial prefrontal cortex (MPFC) (Greicius et al., 2009). Functionally, PCC is predominantly known as a core component of the default mode network (Greicius et al., 2003). However, it is also involved in other networks, such as the dorsal attention network and the executive control network (Leech and Sharp, 2014). Due to the extensive functional importance of the PCC across multiple networks, we hypothesize that it is a key hub node facilitating communication between multiple canonical networks. While the importance of the PCC in widespread communication between numerous brain networks is well documented, little is currently known about the impact of biochemical and structural alterations within the PCC on its functional connectivity with other key areas. Hence, we sought to elicit the impact of local biochemical and structural factors in the PCC on the fRCC in a healthy population.

Proton magnetic resonance spectroscopy (MRS) enables the identification of regionally specific metabolic signatures non-invasively (Duncan, 1996). Of particular importance to the present study, one particular metabolite, *N-acetyl Aspartate* (NAA), can be identified using this technique. NAA is thought to be present predominantly in neuronal cells, which has been shown to be a biomarker for neuronal and axonal integrity (Dautry et al., 2000; Demougeot et al., 2001). Another measure, which can be acquired using T1-weighted structural images allows for the quantification of neuronal and glial density (Li et al., 2014), known as cortical thickness (CTh).

Cortical thinning has been shown to occur due to aging in a healthy population (Salat et al., 2004). Furthermore, decreased cortical thickness within the pregenual cingulate cortex has been observed in patients suffering from late onset depression (Mayberg, 2003; Lim et al., 2012). One recent study identified a positive correlation between CTh and NAA in the dorsal anterior cingulate cortex (Li et al., 2014). However, the relationship between CTh, NAA, and the rich club coefficient are to our knowledge unexplored.

Due to the highly influential nature of the PCC at a functional level, we hypothesized that it exerts a substantial influence on the functional backbone of the network. Furthermore, we conjectured that both local metabolic and structural properties impact on the extent of its influence. Hence, we sought to elicit the impact of local metabolic and structural properties of the PCC on the overall importance of the rich club.

## Materials and methods

Forty eight healthy volunteers (33.08 ± 8.68 years) were recruited in Magdeburg, Germany. The sample consisted of 35 males and 13 females. Participants were excluded based on major medical illness, pregnancy, history of seizures, current psychiatric disorder or general MRI contradictions. The study was approved by the institutional review board of the University of Magdeburg and all subjects gave written informed consent before inclusion.

### Data acquisition

The fMRI data were acquired on a 3 Tesla Siemens MAGNETOM Trio scanner (Siemens, Erlangen, Germany) with an eight-channel phased-array head coil. Subjects were requested to lie still with their eyes closed for the duration of the resting state scan. A total of 488 volumes were acquired with an echo-planar imaging sequence. The following acquisition parameters were used: echo time = 25 ms, field of view = 22 cm, acquisition matrix = 44 × 44, isometric voxel size = 5 × 5 × 5 mm^3^. Whole brain coverage was achieved with 26 contiguous axial slices, using a repetition time of 1,250 ms and flip angle of 70°. The first 10 volumes were discarded to allow for magnetic field homogenization. High resolution T1-weighted structural MRI scans of the brain were acquired for structural reference using a 3D-MPRAGE sequence (TE = 4.77 ms, TR = 2,500 ms, T1 = 1,100 ms, flip angle = 7°, bandwidth = 140 Hz/pixel, acquisition matrix = 256 × 256 × 192, isometric voxel size = 1.0 mm^3^).

### Data preprocessing

Functional data were corrected for differences in slice time acquisition, motion-corrected using a least squares approach and a six-parameter (rigid body) linear transformation and spatially normalized. The data were linearly detrended. All subjects had less than 1 mm head motion in any direction during the scanning session. An additional regression of nuisance covariates was applied during which the functional data was corrected for global mean signal as well as for white matter and cerebrospinal fluid signal and bandpass filtering between the frequencies of 0.01 and 0.1 Hz. Data were preprocessed using spm8 (Wellcome Trust Center for Neuroimaging, London, England) using the data processing assistant for resting-state fMRI (DPARSF version 2.3, Yan and Zang, 2010).

The resulting volumes were parcellated into 102 nodes using a modified version of the automatic anatomic labeling (AAL) atlas (Tzourio-Mazoyer et al., 2002) containing a fine grained parcellation for the cingulate and insular cortices (Horn et al., 2010; Lord et al., 2012). To compute the resting state functional connectivity (rsFC) of the ROIs, the mean time course of every voxel within each ROI was extracted and Pearsons correlation coefficients were calculated pair-wise for all pairs of ROI's, resulting in 102 by 102 symmetric matrices.

### MRS data acquisition and analysis

**Figure 1:**
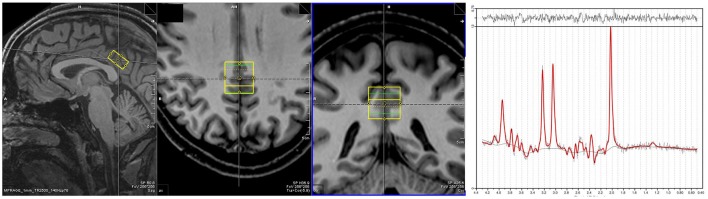
**Placement of MRS voxel in the PCC**. An exemplar spectra for the MRS signal is on the right.

Proton MRS data were acquired from a single voxel (10 × 20 × 20 mm^3^), located in the PCC for each subject at rest (Figure 1). MRS data were acquired using a point resolved spectroscopy (PRESS) sequence with an echo time of 80 ms, repetition time of 2,000 ms, 256 averages, 1,200 Hz bandwidth, 853 ms acquisition time and water suppression. Shimming was performed automatically, with manual fine tuning to improve field homogeneity. To correct for eddy currents, four pulses without water suppression (TR = 10 s) were averaged. Voxel acquisition time was 8 min and 40 s. Spectra were analyzed using LCModel version 6.3.0 (Provencher, 2001). Sixteen different metabolites (Creatine, Glutamate, myo- Inositole, Lactate, NAA, Phosphocholine, Taurine, Aspartate, GABA, Glutamine, Glucose, Alanine, NAAG, Phosphocreatine, Guanine, and Glycerophosphocholine) were fitted using a basis set including all these substances. The concentration of NAA was calculated as its ratio to creatine (Cr) as it is known to be an appropriate reference (Yildiz-Yesiloglu and Ankerst, 2006). Model fit error was estimated using the Cramer-Rao lower bounds (CRLB) (Cavassila et al., 2001) measure, with spectra deemed to be reliable if CRLB was less than 20%. Other minimum quality criteria included a full width half maximum value of smaller than 12 Hz and a signal to noise ratio of greater than eight.

### Cortical thickness

Cortical thickness (CTh) was measured across the whole brain using the CIVET pipeline (Zijdenbos et al., 2002). MR-images were linearly transformed (rigid body, 6 degrees of freedom) to standard Montreal Neurological Institute (MNI) space with intensity and non-uniformity also corrected (Sled et al., 1998). These images were then segmented into gray matter (GM), white matter (WM), cerebral spinal fluid using a neural net classifier. Next cortical surfaces were extracted for both the pial-cortical surface and GM-WM boundary for each hemisphere separately using a deformable mesh model implemented inside the Constrained Laplacian Anatomic Segmentation using Proximity algorithm (June et al., 2005). Both surfaces were non-linearly registered to a high resolution standard template to ensure the co-registration of all subjects to a common space (Lyttelton et al., 2007). To minimize morphological distortion, the inverse of the aforementioned transformation was applied for each subject and CTh was measured in native space. To calculate the GM percentage in the MRS voxel, the voxel was co-registered to the subjects T1 image using SPM8. MRS voxels for each subject were projected into template space using each subject's affine matrix calculated to transform each hemisphere to standard space earlier.

### Rich club coefficient

These matrices were rendered sparse by recursively removing connections, starting with the lowest correlation until only 10% of connections remain. The sparsity of 10% was selected as networks with more edges tends to introduce weaker, noisy effects obscuring between-group effects (Rubinov et al., 2009; Lord et al., 2012; Borchardt et al., 2015). After thresholding no negative edges remained. To ensure that the graph didn't disconnect, edges which would cause a disconnection if removed were retained even if their correlation was below the cutoff threshold. These sparse networks were then binarized, and the degree of each node is calculated.

The fRCC was then calculated across a range of degree thresholds. This was done by removing nodes with a degree of less than the chosen threshold and dividing the number of connections in the remaining subnetwork with the maximum possible number of connections. This ratio in and of itself is known not to be a good measure of interconnectedness (van den Heuvel and Sporns, 2011), thus we reference this information against what we would expect by random chance. To this end, we randomly move connections in the thresholded connectivity matrix, creating a randomized matrix with the same degree distribution (Maslov and Sneppen, 2002), preserving the degree distribution of the network while destroying the connection pattern. The rich club coefficient for each randomized graph was then calculated and the ratio of the raw RCC to the mean RCC of all randomized graphs at each degree threshold was computed. As such, a normalized RCC of 1 speaks to the connectivity pattern of the rich club being equal to a random graph, that is, high degree nodes are no more or less likely to connect to each other than by random chance. A value greater than 1 shows an affinity of rich nodes to cluster more tightly than expected together, while a value below 1 shows rich nodes predominantly connect with non-rich nodes. Since all analysis using the rich club refers to the normalized rich club and not the raw rich club, the normalized rich club will be henceforth described solely as the rich club. The regions included as rich club members were also extracted.

Rich club membership was defined on an individual level. That is, for each individual the degree threshold giving the highest RCC was identified, then the regions identified as members at this threshold were selected. At the group level, rich club members were identified as those regions appearing in at least 50% of subjects.

## Results

**Figure 2:**
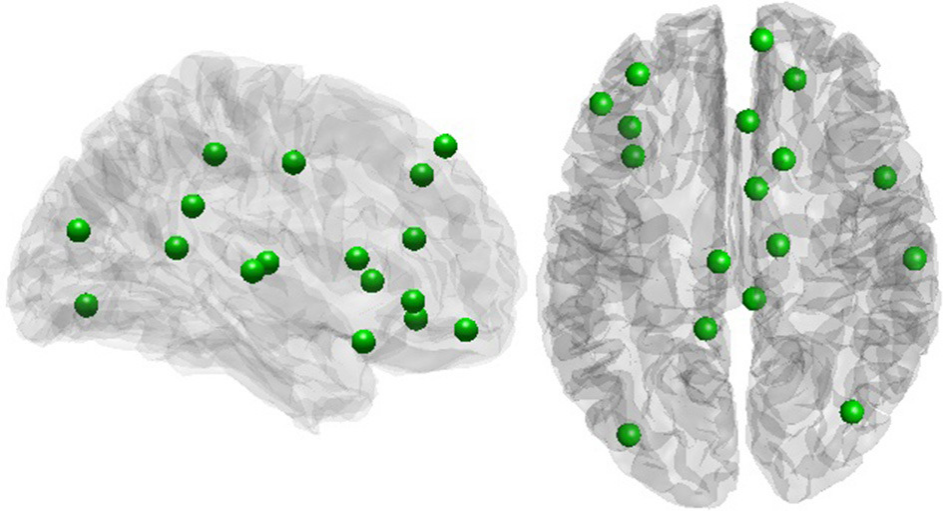
**Rich club members**.

A positive rich club coefficient was observed for all subjects, with the largest fRCC across the group at a degree threshold of 9 (Figure S1). Twenty-one rich club members were consistent in at least half of the participants (Figure 2), with seven out of 21 regions from within the cingulate cortex, including the PCC (Table 1). No correlation between global signal and fRCC was observed (Figure S2).

**Table 1:** **Rich club members**.

**Subjects**	**Region**
45	Frontal Sup Orb R
44	Frontal Mid L
43	Rostral ACC R
43	Pregenual ACC R
42	Temporal Sup R
41	Rostal ACC L
40	Post Insula R
38	Temporal Sup L
37	Thalamus R
36	Temporal Pole Sup L
35	Frontal Inf Tri L
34	Posterior MCC R
33	Caudate R
33	23d L
32	dPCC R
31	Frontal Inf Orb L
31	vPCC L
30	Occipital Mid R
30	Occipital Inf L
28	Medial Prefront Upper R
26	Anterior Insula L

**Figure 3:**
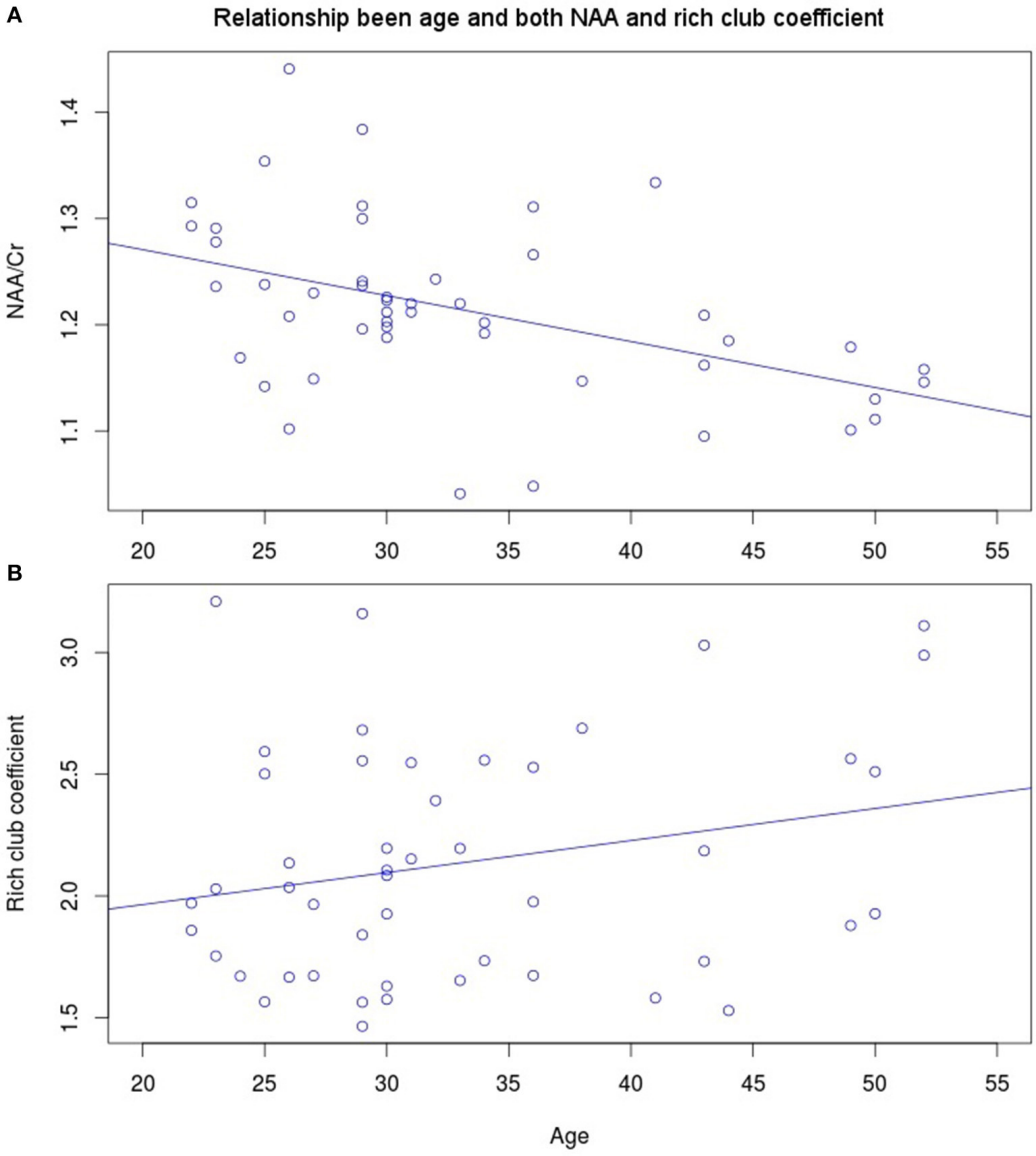
**Linear correlates of age with (A)** NAA (*r* = −0.456, *p* = 0.001, *t* = −3.475, *df* = 46) and **(B)** rich club coefficient (*r* = 0.2356, *p* = 0.107, *t* = 1.644, *df* = 46).

**Figure 4:**
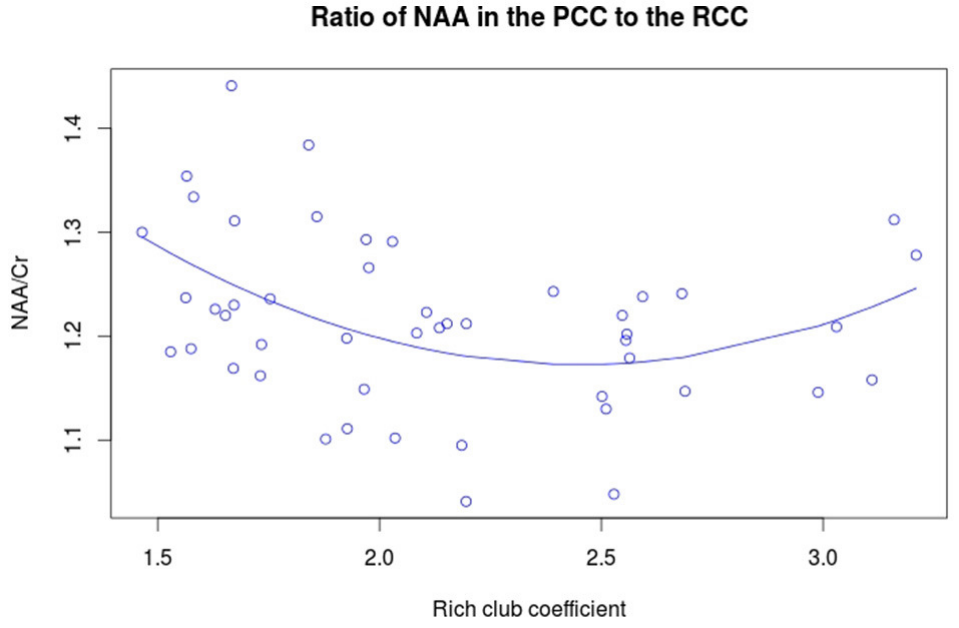
**Quadratic relationship between RCC and NAA/Cr**. The equation for this curve is: *y* = 1.933 – 0.621x + 0.127x^2^ where x is the rich club coefficient.

NAA in the PCC negatively correlated with age (Figure 3A, *r* = −0.456, *p* = 0.001, *t* = −3.475, *df* = 46), while no significant correlation exists between rich club coefficient and age (*r* = 0.2356, *p* = 0.107, *t* = 1.644, *df* = 46, Figure 3B). Furthermore, a significant quadratic relationship was identified between RCC and NAA/Cr in the PCC (Equation 1, Figure 4, *p* = 0.009). This relationship was not observed using a linear model (*r* = −0.041, *p* = 0.093, *t* = −1.708, *df* = 46). The model fit was formally tested using the bayesian information criterion (BIC), which revealed that a quadratic model fit the data better than a linear model (delta BIC = −3.16) or any higher order polynomial (Figure S3). Linear correlates between low and high RCC values were assessed *post hoc*. The data were split into two groups, below the RCC value predicted to give the lowest concentration of NAA/Cr (2.44), and above this value. A negative linear correlation was observed in the low RCC values (*r* = −0.365, *p* = 0.036, *t* = −2.185, *df* = 31), while a trending positive linear correlation was observed in the high RCC values (*r* = 0.473, *p* = 0.075, *t* = 1.935, *df* = 13). Using a Fisher R-to-Z transformation, these two correlations were significantly different (*z* = −2.62, *p* = 0.009, Figure S4). In contrast this did not translate into dependency of global metrics (SWI and CC), which did not correlate with PCC-NAA.

(1) $$\begin{array}{l} \text{Nonlinear relationship between NAA}/\text{Cr in the PCC and}\\ \text{the RCC}.\;\text{F}=5.206,\text{p}=0.009254.\\ \text{y}=1.933-0.621\text{x}+0.127{\text{x}}^{2} \end{array}$$

**Figure 5:**
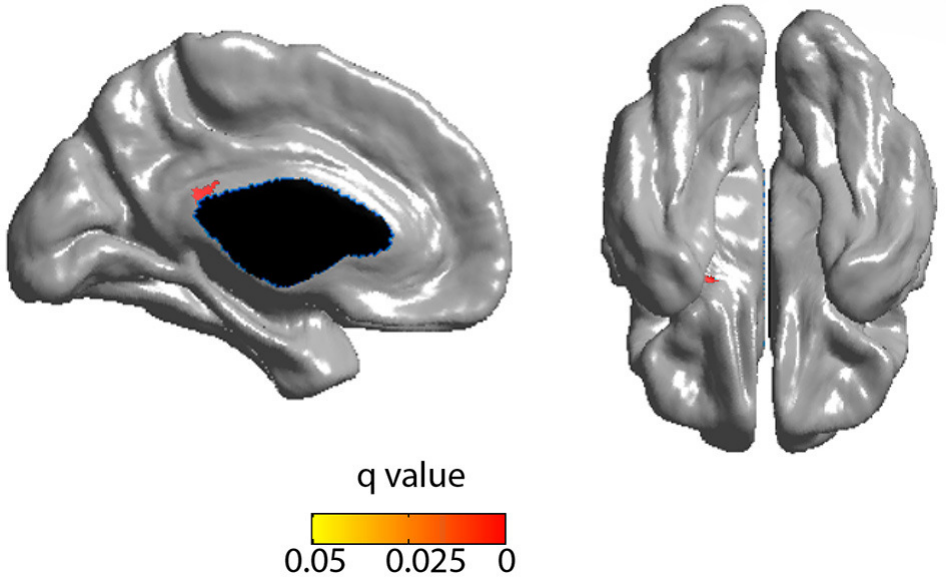
**Positive correlation between RCC and CTh (FDR corrected) incorporating age and NAA as covariates**. Blue represents FDR corrected significant differences. Red/yellow represent FWE corrected significant differences.

**Figure 6:**
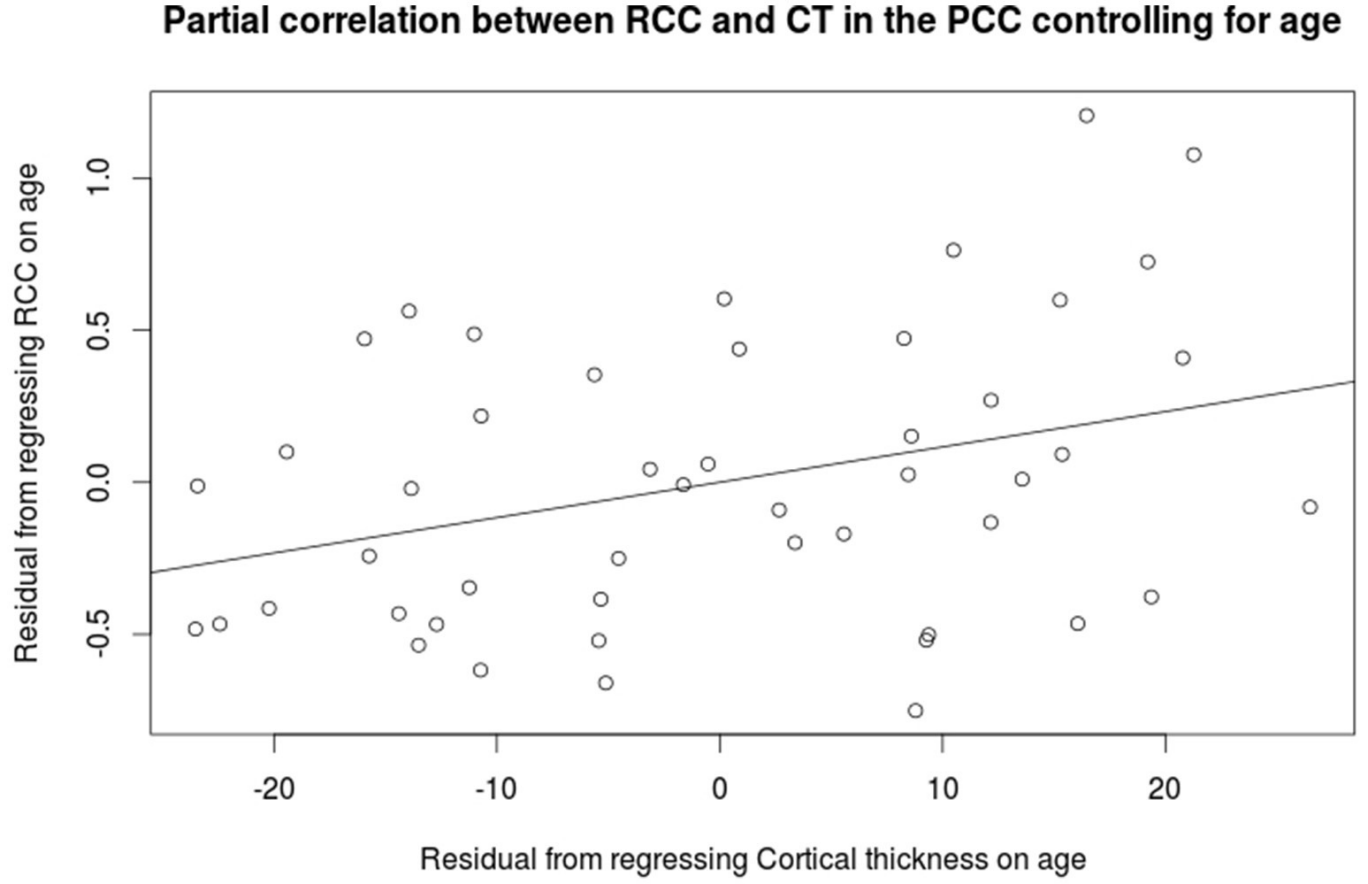
**Partial correlation between the rich club coefficient and mean cortical thickness in the PCC (*r* = 0.339, *p* = 0.015, *t* = 2.519)**.

Cortical thickness in the PCC positively correlated with the rich club coefficient after accounting for age and NAA/Cr as covariates (Figure 5, FDR corrected). Although extensive parts of the cortex showed reduced CT as a factor of age (Figure S5), this did not overlap with the previous finding. CT was also positively correlated with NAA/Cr, predominantly in the mid cingulate cortex, but did not overlap with the fRCC correlation with CT in the PCC (Figure S6). After accounting for age as a covariate, fRCC and mean CTh in the PCC was positively correlated (*r* = 0.339, *p* = 0.015, *t* = 2.519, Figure 6). When considering only age as a covariate, a cluster of vertices in the same location is observed at an uncorrected threshold only (Figure S7).

## Discussion

Although the rich club coefficient has become increasingly studied in both functional and structural networks in human brains (van den Heuvel et al., 2012; Collin et al., 2014b; Grayson et al., 2014), little is currently known about the impact of local metabolic and structural properties of key rich club regions on the integrity of the club as a whole. Hence, we sought to identify effects of neuronal integrity and cortical thinning in the PCC on the density of connections within this functional backbone of the human brain.

Regional distribution of rich club members in this study were in line with previous work (van den Heuvel and Sporns, 2011; van den Heuvel et al., 2013), showing most of the members of the rich club surrounding the inter-hemispheric fissure. Furthermore, we observe many areas of the cingulate cortex as members of the rich club, suggesting a high level of communication both within the cingulate cortex as well as to other regions of the brain. This result speaks to the central role of the cingulate cortex in information transfer at a whole brain level. Indeed, one third of all rich club members were subregions of the cingulate cortex, including pregenual and rostal parts of the ACC, posterior MCC, dorsal and ventral PCC and Brodman area 23d. During preprocessing we regressed out global signal from out resting state data. This procedure has met with criticism as it can introduce spurious relationships within the data. We observed no relationship between fRCC and GSR, suggesting our results are not driven by the global signal.

The quadratic relationship between the functional rich club and NAA/Cr initially declines linearly, then increases at higher RCC levels. This suggests that other factors, in addition to the NAA/Cr concentration in the PCC, influence the functional rich club coefficient. Confirming this, we found a significant reduction of CTh in the PCC related to the RCC, after accounting for age and NAA/Cr. These results show that both the neuronal integrity and cortical thickness affect the richness of the network.

The directionality of correlations will require further in depth analysis, at best including suitable behavioral parameters. Lower NAA is normally considered indicative of reduced neuronal integrity while its relevance to functional rich club alterations is less clear. Age related whole network changes for example in Alzheimers disease found effects rather in peripheral functional connections leaving the core network rather undisturbed (Daianu et al., 2015) at least for functional connectivity properties. In clinical context, schizophrenia was associated with reduced rich club connections, speaking in favor of a beneficial role of higher functional rich club routing and routing efficiency.

Nodes of the structural rich-club are especially resilient to targeted attacks and enhance global information flow by acting as a “highway” system (van den Heuvel and Sporns, 2011; van den Heuvel et al., 2012; Xia et al., 2016). Nevertheless, the structural rich-club is a relatively high-cost component of brain networks because the wiring cost is greater between members of the rich club than between less well-connected nodes in the periphery (Collin et al., 2014b). Also, structural rich-club nodes have higher levels of metabolic energy consumption than peripheral nodes (Collin et al., 2014b). Structural rich clubs were shown to connect early and are maintained through in maturation (“rich-get-richer”), suggesting that these nodes may have a key developmental role (Schroeter et al., 2015). Thus, it has been argued that maintenance of such a costly network component should offer advantages to the brain's computational performance. In line with this thought, mature rich-clubs were indeed shown to be of great importance for routing of spontaneous activity flow in the network frequently acting as brokers for spontaneous multi-unit activity, suggesting a role of rich-clubs for orchestrating coordinated activity in the network, for example switching between different network states (Crossley et al., 2013; Leech and Sharp, 2014; Senden et al., 2014; Schroeter et al., 2015).

In our case we found lower fRCC associated with higher NAA, thus a direct interpretation in terms of functional relevance will be difficult, particularly since we only investigated healthy subjects.

Our results are in line with previous accounts for the influence of local metabolism within a node on brain network configuration. Horn et al. reported that functional resting state connectivity between pgACC and anterior insula was correlated with Glx concentrations in pgACC (Horn et al., 2010). This effect was localized in that insula MRS did not show similar influences on this connectivity. More recently, Demenescu et al. (2016) show that anterior insula MRS predicted long range resting state connectivity, however toward temporo-parietal and visual cortices. Importantly, these findings of glutamatergic modulations were found explicitly for depressed patients, while healthy controls did not show the same correlation (Horn et al., 2010). This was interpreted as a reflection of a primary glutamatergic deficit in MDD, as supported by respective meta-analyses (Taylor, 2014) while in the absence of a direct metabolic deficit variance of the metabolite was considered to be too low.

In contrast, work on PCC MRS in healthy subjects reported an interrelation of both glutamate and GABA on PCC functional connectivity, at least when investigating the default mode network as a whole via independent component analysis (Kapogiannis et al., 2013). Therefore, it is plausible that also in healthy conditions, variance of both functional connectivity and brain metabolites as acquired in MRS is large enough to detect covariations in healthy cohorts. Such covariations should then reflect a common biological mechanism and the most prominent mechanism in healthy cohorts would be that of aging.

Age related brain changes have been investigated in greater extent and prominent effect have ben postulated for NAA (Block et al., 2002). Indeed we found NAA to be correlated with age and RCC however there was no direct correlation between RCC and age. Furthermore, the relationship of PCC cortical thickness was controlled for potential age effects, thus pointing toward a second, age independent mechanism, which relates PCC functional integrity as evident in cortical thickness and NAA levels toward RCC. It must also be stated that the sample included in our study is of rather young age and thus neurodegenerative effects, previously investigated may not be the main driver.

Although Grayson et al. (2014) found an enriched rich club in adolescents, we did not see a significant correlation between age and fRCC (*p* = 0.1068), which may be explained by the vast developmental changes in the brain during the adolescent period (Giedd, 2004) in comparison the putative effects in our sample.

An alternative interpretation would consider the mild apparent, if not statistically significant increase of fRCC with age as a subtle counterpart to the significant age dependent NAA decrease. As such MRS would be considered a very sensitive age marker while secondary effects of age on whole brain organization would be more subtle and not yet detectable in the observed age range between early twenties and mid-fifties. This would then be reflected by the linear component fRCC increase with decreasing NAA, and may, in a far stretch, be interpreted as a sign of age related “hyper-efficiency.”

However, such interpretation would not work for the observed quadratic relationship, which for brains with very high RCC's rather found the inverse relationship of increasing fRCC with higher NAA. This suggests that other factors, in addition to the NAA/Cr concentration in the PCC, influence the rich club coefficient. In support of this interpretation, one may add that we found linear negative relationship of CTh in the PCC and fRCC. However, we found this after accounting for age and NAA/Cr, therefore results at this stage should best be appreciated in that we found evidence that both the neuronal integrity and cortical thickness affect the richness of the network and the parallel, potentially driving, or counteracting effects of age and other sources of inter-individual variation will be subject to future investigations in larger cohorts.

Within the context of these considerations, our results in a comparably large, young adult population point toward an effect of local neuronal integrity or at least neurobiological constitution on resting state network configuration.

The new advance in our finding is first the extension to NAA, after previous reports mainly focused on neurotransmitters GABA and Glutamate. Secondly after previous research as investigated metabolite influence on direct connections of a region, either in terms of seed based edges or in terms of the independent component which hosts the location of the single MRS voxel, we extend the view toward a much more global effect on network constitution. This effect is very likely due to the central role of the PCC within the rich club. While we cannot test this explicitly at this stage, we would assume that the relationship found here is mainly representative of the central role of the PCC within a backbone of small network organization rather than due to the specific impact of NAA in comparison to GABA or glutamate. Ideally, to test such claim, one would request MRS sequences which allow assessment of all mentioned metabolites within the scope of a single session along a number of regions and further leave time for additional resting state assessments. Such methods are available for higher field strengths, making explicit use of the improve line separation (Dou et al., 2013; Li et al, 2016).

## Limitations

Only 13 out of 48 subjects included in this study were female. Due to the small sample of females included, gender effects have not been discussed. However, future work would benefit from investigating gender specific variation in the context of NAA, CTh and RCC.

## Conclusion

The rich club in fMRI is an area of growing interest, with implications in neurological development (Grayson et al., 2014) and psychiatric disease (van den Heuvel et al., 2013; Collin et al., 2014a). Our findings add evidence to this growing avenue of research, progressing our understanding of the impact of metabolic and structural factors on the rich club. To our knowledge, this is the first study identifying the impact of these factors targeted to the PCC, showing a relationship between CT and NAA/Cr neurotransmitter levels and its effects on this core subnetwork of resting state functional connectivity via the PCC.

## Author contributions

MW and HH designed the experiment. AK, MW, and ML acquired the data. AL, ML, MW, Jv, LRD, and VB analyzed the data. AL, VB, MW, and MB interpreted the results. AL drafted the manuscript. AL, VB, MW, ML, Jv, and LRD revised the manuscript. All authors approved the final version of the manuscript.

## Funding

This study was supported by funding from the following grants: SFB 779/A6 (German Research Foundation) to MW and FP7 MC-ITN R'Birth (Marie Curie) to AL and MW.

### Conflict of interest statement

The authors declare that the research was conducted in the absence of any commercial or financial relationships that could be construed as a potential conflict of interest.

## References

[B1] Block W. Träber F. Flacke S. Jessen F. Pohl C. Schild H. (2002). *In-vivo* proton MR-spectroscopy of the human brain: assessment of N-acetylaspartate (NAA) reduction as a marker for neurodegeneration. Amino Acids 23, 317–323. 10.1007/s00726-001-0144-0 12373553

[B2] Borchardt V. Krause A. L. Starck T. Nissilä J. Timonen M. Kiviniemi V.. (2015). Graph theory reveals hyper-functionality in visual cortices of seasonal affective disorder patients. World J. Biol. Psychiatry 16, 123–134. 10.3109/15622975.2014.966144 25363311

[B3] Buckner R. L. Bandettini P. A. O'Craven K. M. Savoy R. L. Petersen S. E. Raichle M. E.. (1996). Detection of cortical activation during averaged single trials of a cognitive task using functional magnetic resonance imaging. Proc. Natl. Acad. Sci. U.S.A. 93, 14878–14883. 10.1073/pnas.93.25.14878 8962149 PMC26230

[B4] Calhoun V. D. Adali T. McGinty V. B. Pekar J. J. Watson T. D. Pearlson G. D. (2001). fMRI activation in a visual-perception task: network of areas detected using the general linear model and independent components analysis. Neuroimage 14, 1080–1088. 10.1006/nimg.2001.0921 11697939

[B5] Cavassila S. Deval S. Huegen C. van Ormondt D. Graveron-Demilly D. (2001). Cramér-Rao bounds: an evaluation tool for quantitation. NMR Biomed. 14, 278–283. 10.1002/nbm.701 11410946

[B6] Colizza V. Flammini A. Serrano M. A. Vespignani A. (2006). Detecting rich-club ordering in complex networks. Nat. Phys. 2, 110–115. 10.1038/nphys209

[B7] Collin G. Kahn R. S. de Reus M. A. Cahn W. van den Heuvel M. P. (2014a). Impaired rich club connectivity in unaffected siblings of schizophrenia patients. Schizophr. Bull. 40, 438–448. 10.1093/schbul/sbt162 24298172 PMC3932089

[B8] Collin G. Sporns O. Mandl R. C. W. van den Heuvel M. P. (2014b). Structural and functional aspects relating to cost and benefit of rich club organization in the human cerebral cortex. Cereb. Cortex. 24, 2258–2267. 10.1093/cercor/bht064 23551922 PMC4128699

[B9] Crossley N. A. Mechelli A. Vértes P. E. Winton-Brown T. T. Patel A. X. Ginestet C. E.. (2013). Cognitive relevance of the community structure of the human brain functional coactivation network. Proc. Natl. Acad. Sci. U.S.A. 110, 11583–11588. 10.1073/pnas.1220826110 23798414 PMC3710853

[B10] Daianu M. Jahanshad N. Mendez M. F. Bartzokis G. Jimenez E. E. Thompson P. M. (2015). Communication of brain network core connections altered in behavioral variant frontotemporal dementia but possibly preserved in early-onset Alzheimer's disease. Proc. SPIE Int. Soc. Opt. Eng. 9413:941322. 10.1117/12.2082352 25848494 PMC4384394

[B11] Damoiseaux J. S. Rombouts S. A. R. B. Barkhof F. Scheltens P. Stam C. J. Smith S. M.. (2006). Consistent resting-state networks across healthy subjects. Proc. Natl. Acad. Sci. U.S.A. 103, 13848–13853. 10.1073/pnas.0601417103 16945915 PMC1564249

[B12] Dautry C. Vaufrey F. Brouillet E. Bizat N. Henry P. G. Condé F.. (2000). Early N-acetylaspartate depletion is a marker of neuronal dysfunction in rats and primates chronically treated with the mitochondrial toxin 3-nitropropionic acid. J. Cereb. Blood Flow Metab. 20, 789–799. 10.1097/00004647-200005000-00005 10826529

[B13] Demenescu L. R. Colic L. Li M. Safron A. Biswal B. Metzger C. D.. (2016). A spectroscopic approach toward depression diagnosis: local metabolism meets functional connectivity. Eur. Arch. Psychiatry Clin. Neurosci. 267, 95–105. 10.1007/s00406-016-0726-1 27561792

[B14] Demougeot C. Garnier P. Mossiat C. Bertrand N. Giroud M. Beley A.. (2001). N-Acetylaspartate, a marker of both cellular dysfunction and neuronal loss: its relevance to studies of acute brain injury. J. Neurochem. 77, 408–415. 10.1046/j.1471-4159.2001.00285.x 11299303

[B15] Dou W. Palomero-Gallagher N. van Tol M.-J. Kaufmann J. Zhong K. Bernstein H.-G.. (2013). Systematic regional variations of GABA, glutamine, and glutamate concentrations follow receptor fingerprints of human cingulate cortex. J. Neurosci. 33, 12698–12704. 10.1523/JNEUROSCI.1758-13.2013 23904606 PMC6618546

[B16] Duncan J. S. (1996). Magnetic resonance spectroscopy. Epilepsia 37, 598–605. 10.1111/j.1528-1157.1996.tb00622.x 8681890

[B17] Giedd J. N. (2004). Structural magnetic resonance imaging of the adolescent brain. Ann. N.Y. Acad. Sci. 1021, 77–85. 10.1196/annals.1308.009 15251877

[B18] Grayson D. S. Ray S. Carpenter S. Iyer S. Dias T. G. C. Stevens C.. (2014). Structural and functional rich club organization of the brain in children and adults. PLoS ONE 9:e88297. 10.1371/journal.pone.0088297 24505468 PMC3915050

[B19] Greicius M. D. Krasnow B. Reiss A. L. Menon V. (2003). Functional connectivity in the resting brain: a network analysis of the default mode hypothesis. Proc. Natl. Acad. Sci. U.S.A. 100, 253–258. 10.1073/pnas.0135058100 12506194 PMC140943

[B20] Greicius M. D. Supekar K. Menon V. Dougherty R. F. (2009). Resting-state functional connectivity reflects structural connectivity in the default mode network. Cereb. Cortex 19, 72–78. 10.1093/cercor/bhn059 18403396 PMC2605172

[B21] Horn D. I. Yu C. Steiner J. Buchmann J. Kaufmann J. Osoba A.. (2010). Glutamatergic and resting-state functional connectivity correlates of severity in major depression - the role of pregenual anterior cingulate cortex and anterior insula. Front. Syst. Neurosci. 4:33. 10.3389/fnsys.2010.00033 20700385 PMC2914530

[B22] June S. K. Singh V. Jun K. L. Lerch J. Ad-Dab'bagh Y. MacDonald D. (2005). Automated 3-D extraction and evaluation of the inner and outer cortical surfaces using a Laplacian map and partial volume effect classification. Neuroimage 27, 210–221. 10.1016/j.neuroimage.2005.03.036 15896981

[B23] Kapogiannis D. Reiter D. A. Willette A. A. Mattson M. P. (2013). Posteromedial cortex glutamate and GABA predict intrinsic functional connectivity of the default mode network. Neuroimage 64, 112–119. 10.1016/j.neuroimage.2012.09.029 23000786 PMC3801193

[B24] Karni A. Meyer G. Jezzard P. Adams M. M. Turner R. Ungerleider L. G. (1995). Functional MRI evidence for adult motor cortex plasticity during motor skill learning. Nature 377, 155–158. 10.1038/377155a0 7675082

[B25] Langford E. Schwertman N. Owens M. Langford E. Schwertman N. Owens M. (2001). Is the property of being positively correlated transitive? Am. Stat. 55, 322–325. 10.1198/000313001753272286

[B26] Leech R. Sharp D. J. (2014). The role of the posterior cingulate cortex in cognition and disease. Brain 137, 12–32. 10.1093/brain/awt162 23869106 PMC3891440

[B27] Li M. Demenescu L. R. Colic L. Metzger C. D. Heinze H. J. Steiner J.. (2016). Temporal dynamics of antidepressant ketamine effects on glutamine cycling follow regional fingerprints of AMPA and NMDA receptor densities. Neuropsychopharmacology 10.1038/npp.2016.184. [Epub ahead of print]. 27604568 PMC5437874

[B28] Li M. Metzger C. D. Li W. Safron A. van Tol M.-J. Lord A. (2014). Dissociation of glutamate and cortical thickness is restricted to regions subserving trait but not state markers in major depressive disorder. J. Affect. Disord. 169C, 91–100. 10.1016/j.jad.2014.08.001 25173431

[B29] Lim H. K. Jung W. S. Ahn K. J. Won W. Y. Hahn C. Lee S. Y.. (2012). Regional cortical thickness and subcortical volume changes are associated with cognitive impairments in the drug-naive patients with late-onset depression. Neuropsychopharmacology 37, 838–849. 10.1038/npp.2011.264 22048467 PMC3260976

[B30] Lord A. Horn D. Breakspear M. Walter M. (2012). Changes in community structure of resting state functional connectivity in unipolar depression. PLoS ONE 7:e41282. 10.1371/journal.pone.0041282 22916105 PMC3423402

[B31] Lyttelton O. Boucher M. Robbins S. Evans A. (2007). An unbiased iterative group registration template for cortical surface analysis. Neuroimage 34, 1535–1544. 10.1016/j.neuroimage.2006.10.041 17188895

[B32] Maslov S. Sneppen K. (2002). Specificity and stability in topology of protein networks. Science 296, 910–913. 10.1126/science.1065103 11988575

[B33] Mayberg H. S. (2003). Modulating dysfunctional limbic-cortical circuits in depression: towards development of brain-based algorithms for diagnosis and optimised treatment. Br. Med. Bull. 65, 193–207. 10.1093/bmb/65.1.193 12697626

[B34] Meunier D. Lambiotte R. Bullmore E. T. (2010). Modular and hierarchically modular organization of brain networks. Front. Neurosci. 4:200. 10.3389/fnins.2010.00200 21151783 PMC3000003

[B35] Meunier D. Lambiotte R. Fornito A. Ersche K. D. Bullmore E. T. (2009). Hierarchical modularity in human brain functional networks. Front. Neuroinformatics 3:37. 10.3389/neuro.11.037.2009 19949480 PMC2784301

[B36] Provencher S. W. (2001). Automatic quantitation of localized *in vivo* 1H spectra with LCModel. NMR Biomed. 14, 260–264. 10.1002/nbm.698 11410943

[B37] Rubinov M. Knock S. A. Stam C. J. Micheloyannis S. Harris A. W. Williams L. M.. (2009). Small-world properties of nonlinear brain activity in schizophrenia. Hum. Brain Mapp. 30, 403–416. 10.1002/hbm.20517 18072237 PMC6871165

[B38] Salat D. H. Buckner R. L. Snyder A. Z. Greve D. N. Desikan R. S. R. Busa E.. (2004). Thinning of the cerebral cortex in aging. Cereb. Cortex 14, 721–730. 10.1093/cercor/bhh032 15054051

[B39] Schroeter M. S. Charlesworth P. Kitzbichler M. G. Paulsen O. Bullmore E. T. (2015). Emergence of rich-club topology and coordinated dynamics in development of hippocampal functional networks *in vitro*. J. Neurosci. 35, 5459–5470. 10.1523/JNEUROSCI.4259-14.2015 25855164 PMC4388914

[B40] Senden M. Deco G. De Reus M. A. Goebel R. Van Den Heuvel M. P. (2014). Rich club organization supports a diverse set of functional network configurations. Neuroimage 96, 174–182. 10.1016/j.neuroimage.2014.03.066 24699017

[B41] Sled J. G. Zijdenbos A. P. Evans A. C. (1998). A nonparametric method for automatic correction of intensity nonuniformity in MRI data. IEEE Trans. Med. Imaging 17, 87–97. 10.1109/42.668698 9617910

[B42] Sporns O. Honey C. J. Kotter R. (2007). Identification and classification of hubs in brain networks. PLoS ONE 2:e1049. 10.1371/journal.pone.0001049 17940613 PMC2013941

[B43] Taylor M. J. (2014). Could glutamate spectroscopy differentiate bipolar depression from unipolar? J. Affect. Disord. 167, 80–84. 10.1016/j.jad.2014.05.019 25082118

[B44] Tzourio-Mazoyer N. Landeau B. Papathanassiou D. Crivello F. Etard O. Delcroix N.. (2002). Automated anatomical labeling of activations in SPM using a macroscopic anatomical parcellation of the MNI MRI single-subject brain. Neuroimage 15, 273–289. 10.1006/nimg.2001.0978 11771995

[B45] van den Heuvel M. P. Kahn R. S. Goi J. Sporns O. (2012). High-cost, high-capacity backbone for global brain communication. Proc. Natl. Acad. Sci. U.S.A. 109, 11372–11377. 10.1073/pnas.1203593109 22711833 PMC3396547

[B46] van den Heuvel M. P. Mandl R. C. W. Kahn R. S. Hulshoff Pol H. E. (2009). Functionally linked resting-state networks reflect the underlying structural connectivity architecture of the human brain. Hum. Brain Mapp. 30, 3127–3141. 10.1002/hbm.20737 19235882 PMC6870902

[B47] van den Heuvel M. P. Sporns O. (2011). Rich-club organization of the human connectome. J. Neurosci. 31, 15775–15786. 10.1523/JNEUROSCI.3539-11.2011 22049421 PMC6623027

[B48] van den Heuvel M. P. Sporns O. Collin G. Scheewe T. Mandl R. C. W. Cahn W.. (2013). Abnormal rich club organization and functional brain dynamics in schizophrenia. JAMA Psychiatry 70, 783–792. 10.1001/jamapsychiatry.2013.1328 23739835

[B49] Xia M. Lin Q. Bi Y. He Y. (2016). Connectomic insights into topologically centralized network edges and relevant motifs in the human brain. Front. Hum. Neurosci. 10:158. 10.3389/fnhum.2016.00158 27148015 PMC4835491

[B50] Yan C.-G. Zang Y.-F. (2010). DPARSF: a MATLAB toolbox for pipeline” data analysis of resting-state fMRI. Front. Syst. Neurosci. 4:13 10.3389/fnsys.2010.00013 20577591 PMC2889691

[B51] Yildiz-Yesiloglu A. Ankerst D. P. (2006). Review of 1H magnetic resonance spectroscopy findings in major depressive disorder: a meta-analysis. Psychiatry Res. 147, 1–25. 10.1016/j.pscychresns.2005.12.004 16806850

[B52] Zhang D. Y. Raichle M. E. (2010). Disease and the brain's dark energy. Nat. Rev. Neurol. 6, 15–28. 10.1038/nrneurol.2009.198 20057496

[B53] Zijdenbos A. P. Forghani R. Evans A. C. (2002). Automatic “pipeline” analysis of 3-D MRI data for clinical trials: application to multiple sclerosis. IEEE Trans. Med. Imaging 21, 1280–1291. 10.1109/TMI.2002.806283 12585710

