# Supplementary material for: Richness in Functional Connectivity Depends on the Neuronal Integrity within the Posterior Cingulate Cortex
Source: Front Neurosci. 2017 Apr 7;11:184. doi: 10.3389/fnins.2017.00184 (PMC5384321; doi:10.3389/fnins.2017.00184)
Supplement: Supplementary file 1 [file Presentation1.PDF]

Richness in functional connectivity depends on the neuronal integrity within the posterior cingulate cortex

Supplementary information

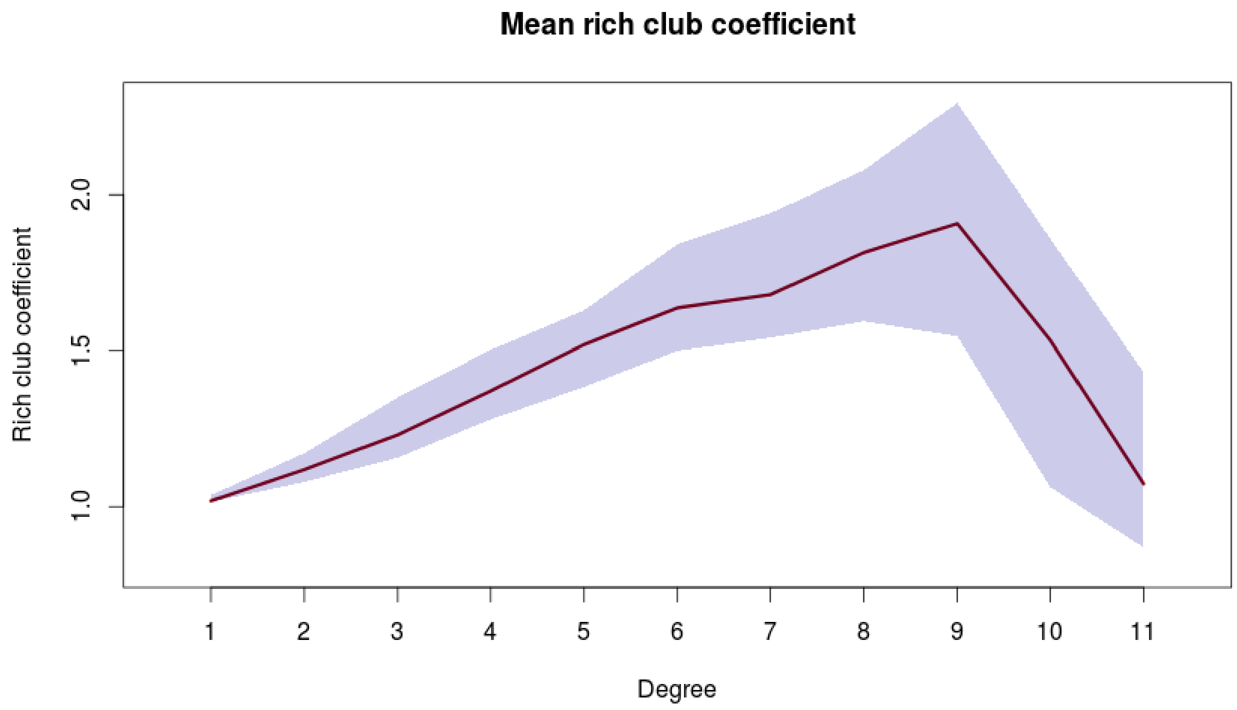

Figure 1: Median rich club coefficient for all subjects. Shaded area represents the area encompassed by Q1 and Q3.

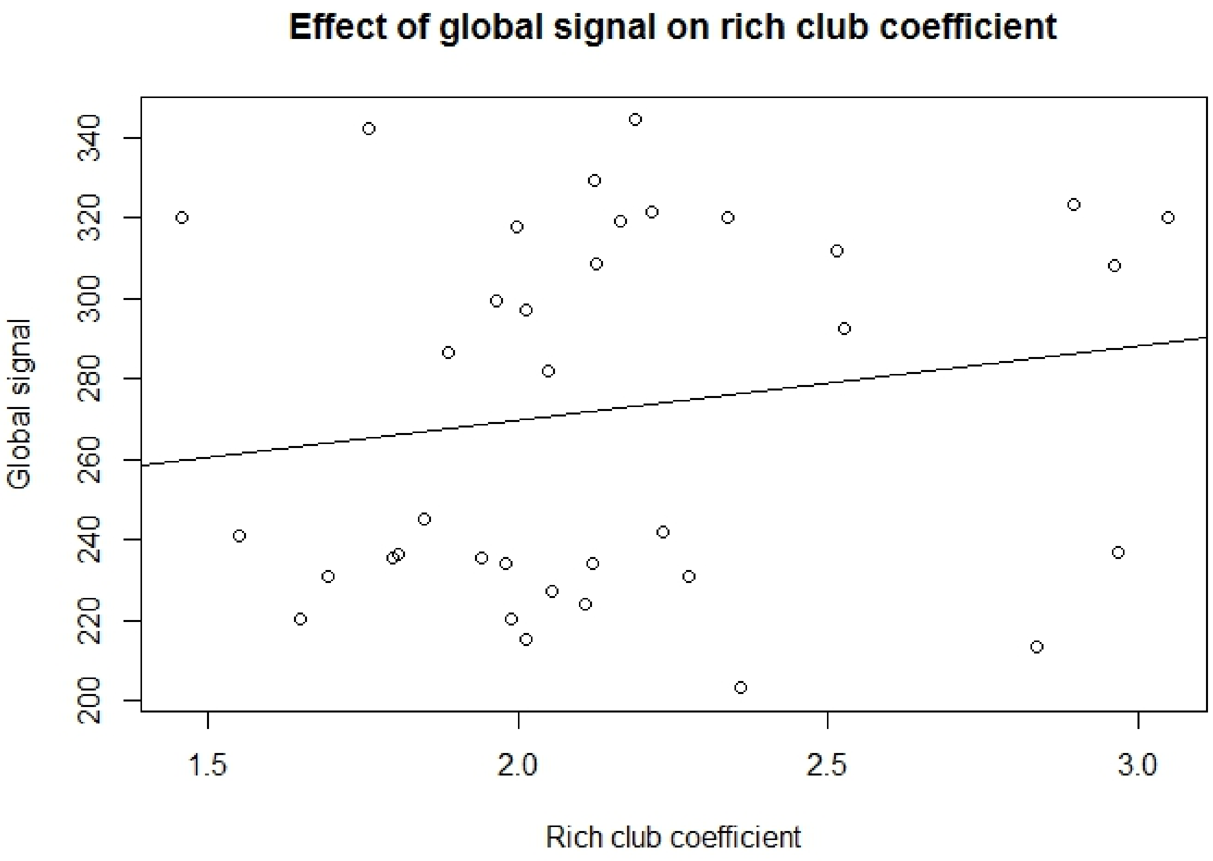

Figure 2: Correlation between Global signal and rich club coefficient. r = 0.165, t = 1.018, p = 0.315, df = 46

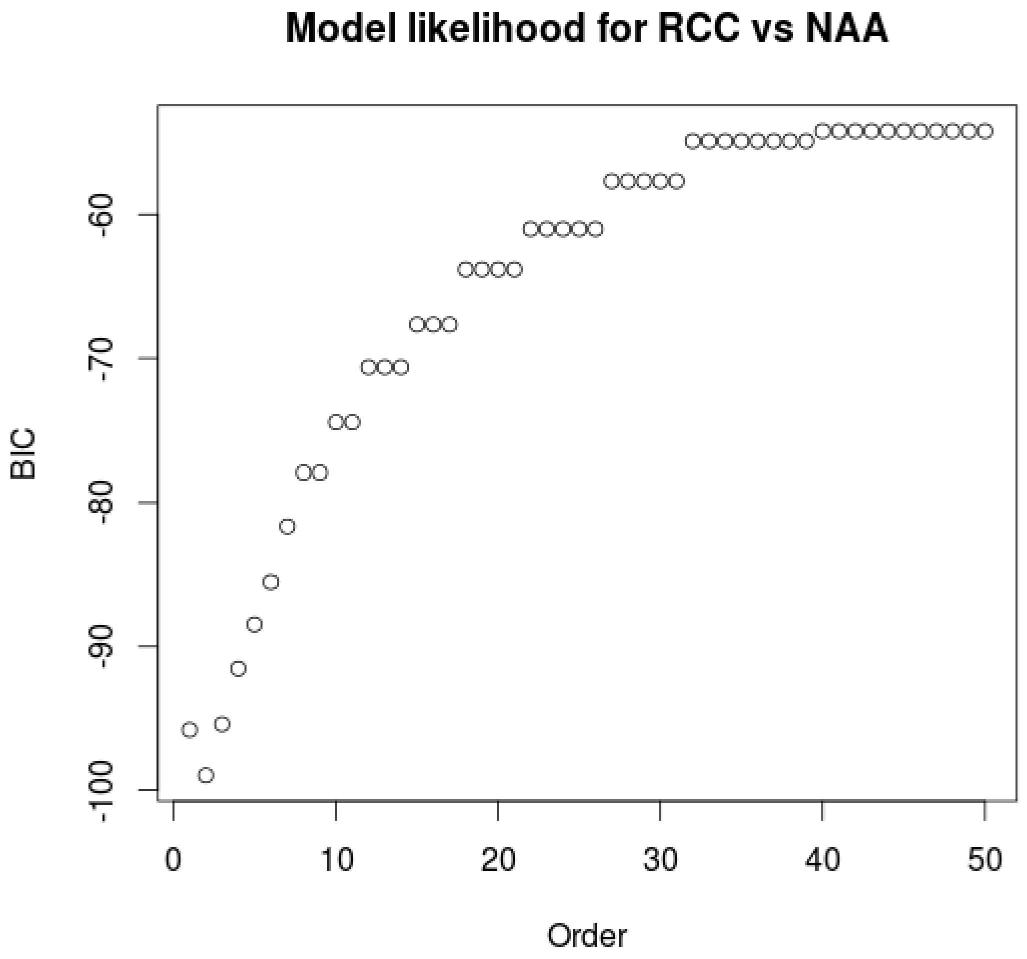

Figure 3: Bayesian information criterion estimates for polynomial models of the RCC and NAA relationship

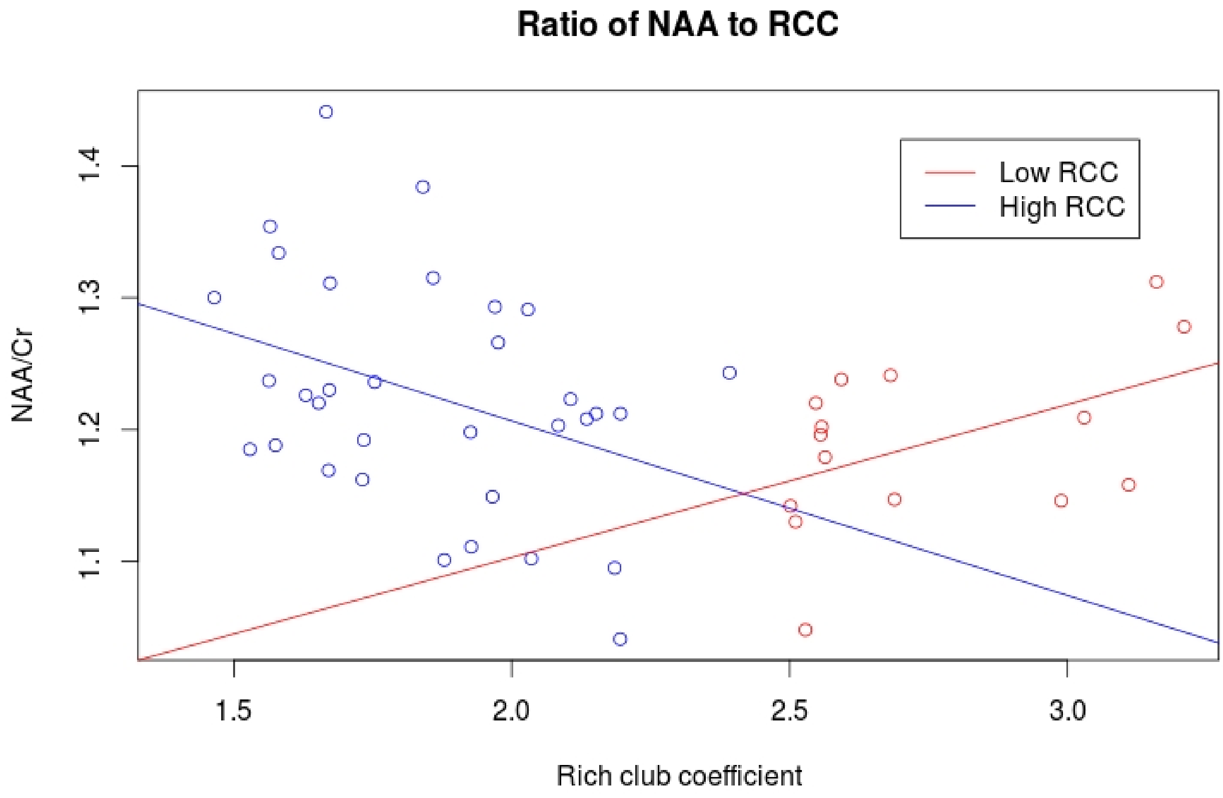

Figure 4: Linear correlates between Low and high rich club coefficients with NAA/Cr. Low RCC: r = -0.365, p = 0.036, t = -2.185, df = 31; High RCC: r = 0.473, p = 0.075, t = 1.935, df = 13; Fisher Z: z = -2.62, p = 0.009

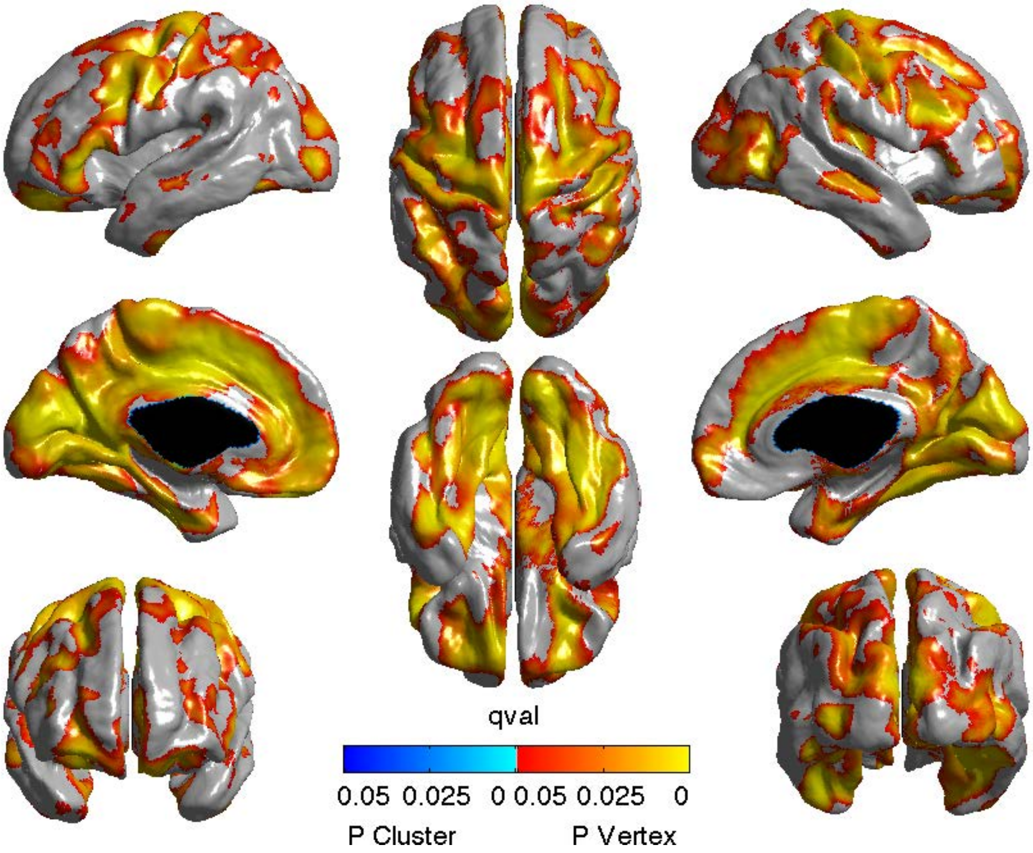

Figure 5: Negative correlation between age and cortical thickness

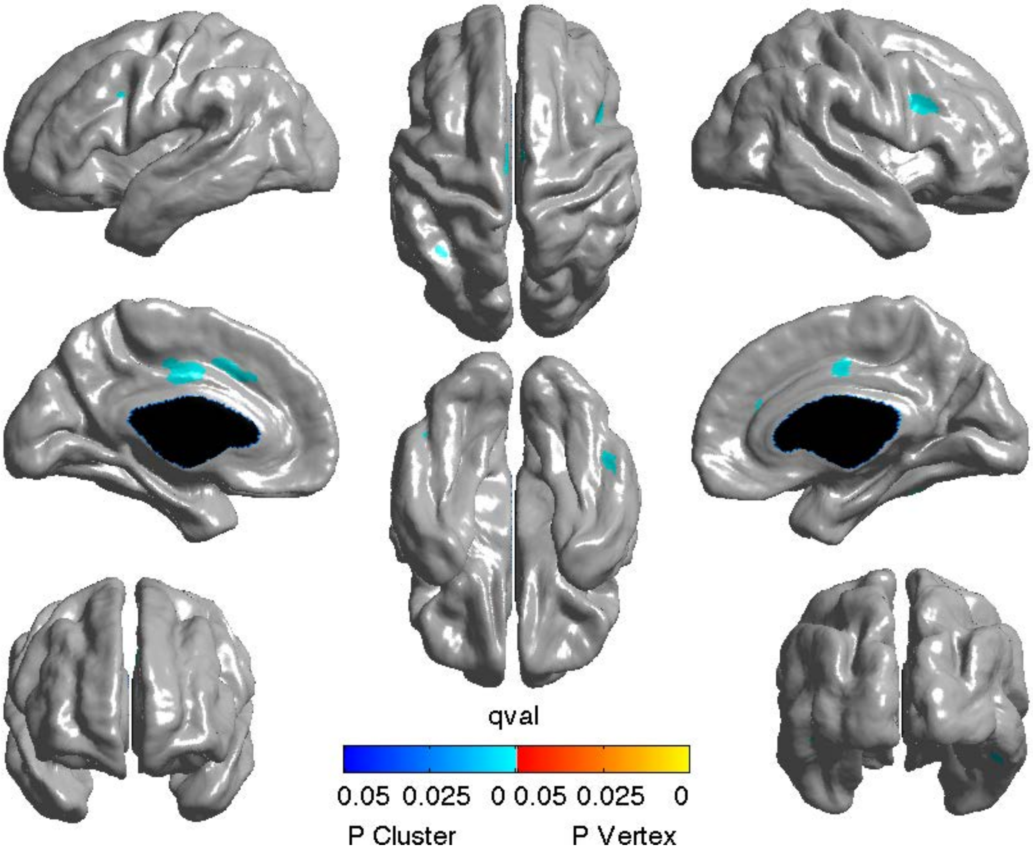

Figure 6: Positive correlation between cortical thickness and NAA/Cr

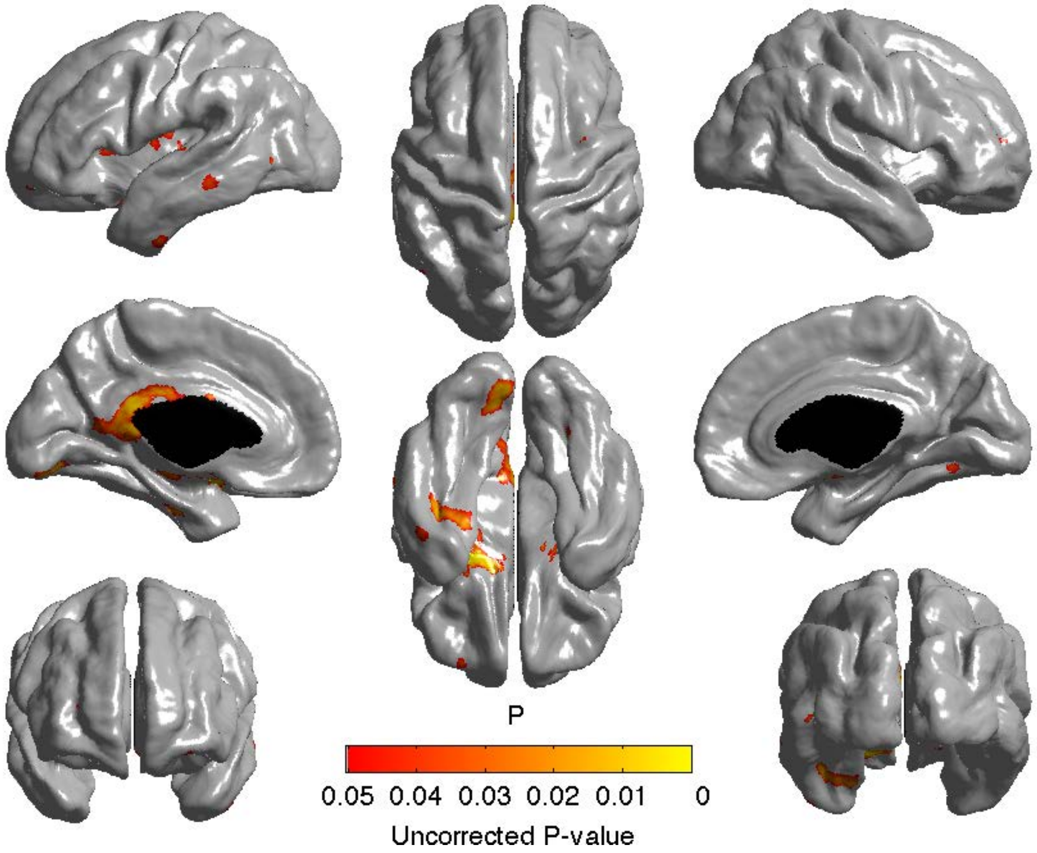

Figure 7: Positive correlation between cortical thickness and the rich club coefficient, accounting for age
